# Stable and efficient CsPbI_3_ quantum-dot light-emitting diodes with strong quantum confinement

**DOI:** 10.1038/s41467-024-50022-8

**Published:** 2024-07-07

**Authors:** Yanming Li, Ming Deng, Xuanyu Zhang, Ting Xu, Ximeng Wang, Zhiwei Yao, Qiangqiang Wang, Lei Qian, Chaoyu Xiang

**Affiliations:** 1 Laboratory of Advanced Nano-Optoelectronic Materials and Devices, Ningbo Institute of Materials Technology and Engineering, Chinese Academy of Science, Ningbo, Zhejiang 315201 China; 2 Laboratory of Advanced Nano-Optoelectronic Materials and Devices, Qianwan Institute of CNITECH, Ningbo, P. R. China, Ningbo, 315300 China; 3 Ningbo University, Ningbo, Zhejiang 315211 China; 4 University of Nottingham Ningbo China, Ningbo, 315100 China; 5 Institute of Information Technology, Shenzhen Institute of Information Technology, Shenzhen, China; 6 Department of Engineering Physics, University of Wisconsin-Madison, Madison, WI 53706 USA; 7 Zhejiang Provincial Engineering Research Center of Energy Optoelectronic Materials and Devices, Ningbo Institute of Materials Technology and Engineering, Chinese Academy of Science, Ningbo, Zhejiang 315201 China

**Keywords:** Quantum dots, Lasers, LEDs and light sources

## Abstract

Even though lead halide perovskite has been demonstrated as a promising optoelectronic material for next-generation display applications, achieving high-efficiency and stable pure-red (620~635 nm) emission to cover the full visible wavelength is still challenging. Here, we report perovskite light-emitting diodes emitting pure-red light at 628 nm achieving high external quantum efficiencies of 26.04%. The performance is attributed to successful synthesizing strongly confined CsPbI_3_ quantum dots with good stability. The strong binding 2-naphthalene sulfonic acid ligands are introduced after nucleation to suppress Ostwald ripening, meanwhile, ammonium hexafluorophosphate exchanges long chain ligands and avoids regrowth by strong binding during the purification process. Both ligands enhance the charge transport ability of CsPbI_3_ quantum dots. The state-of-the-art synthesis of pure red CsPbI_3_ quantum dots achieves 94% high quantum efficiency, which can maintain over 80% after 50 days, providing a method for synthesizing stable strong confined perovskite quantum dots.

## Abstract (web summary)

Li et al. report the regulation of perovskite quantum dot growth after nucleation by designing ligand exchange utilising proton transfer. The Ostwald ripening and regrowth are prohibited with an external quantum efficiency of 26.04% achieved in the pure-red perovskite light-emitting diodes.

## Introduction

Metal halide perovskites (MHPs) are potential materials for next-generation light-emitting diodes (LEDs) because of their excellent properties, such as adjustable band gap, high color purity, high photoluminescence quantum yield (PLQY) and cost-effective solution process^1–4^. To date, tremendous efforts have been applied and high external quantum efficiency (EQE) was obtained in green and crimson (wavelength length is about 670–690 nm) perovskite light-emitting diodes (PeLEDs)^5–8^. However, pure-red PeLEDs with emission wavelength less than 635 nm, which can meet the requirement of Rec.2020, are still inferior to the counterparts mentioned above^9–11^.

To achieve pure red emission, several methods have been studied. Adjusting the ratio of halogen elements such as Br to I makes it easy to achieve pure red PeLEDs. However, the phase separation of mixed halogen CsPbI_3-x_Br_x_ perovskite materials occurs under the excitation of light or the applied voltage, resulting in spectral redshift and performance degradation of PeLED^12,13^. While the low-dimensional phase of quasi-2D perovskite of pure CsPbI_3_ is expected to achieve pure red emission, the wide distribution of multiphase in perovskite thin films leads to inefficient energy transfer and unsatisfactory color purity^14^. Thus, CsPbI_3_ quantum dots (QDs) with strong quantum confinement are expected to achieve wide band gap pure red emission, which can avoid halide separation and multiphase blending, as well as increase the exciton binding energy to improve luminescent efficiency^15^.

Cubic phase (α) CsPbI_3_ perovskite has a narrow band gap (*E*g = 1.73 eV)^16^. CsPbI_3_ QDs that follow classical thermodynamic equilibrium synthesis usually have weak quantum confinement and emission in the crimson region. It is challenging to synthesize CsPbI_3_ QDs with strong quantum confinement to realize pure red emission, where the radius of QDs is less than 5 nm. Small QDs have high surface energy, which is far from equilibrium and easy to grow spontaneously^17^. In the traditional thermal injection method, the rapid nucleation of perovskite QDs is accompanied by the growth of QDs through Ostwald ripening^18^. The traditional weak binding ligands, such as oleic acid (OA) and oleylamine (OAm), are easily debonding, leading to the exposure of the highly active perovskite ionic sites^19,20^. After the monomer in the reaction is exhausted, those active sites accelerate the dissolution of small QDs and the growth of large QDs, increasing the average size of the system and the defocusing of the size distribution^21^. Therefore, uncontrollable Ostwald ripening growth of QDs weakens the quantum confinement effect, and the debonding of weak ligands increases the non-radiative composite defects on the surface of QDs and reduces the stability of QDs. Although CsPbI_3_ QDs can be stabilized by doping smaller metal cations, such as Mn^2+^, and Sr^2+^, to shrink the lattice, it is still difficult to achieve emissions below 640 nm^22,23^. The strong binding ligands or multi-anchored ligands introduced by in situ QDs nucleation or ligand exchange after ripening can significantly improve the durability and stability of CsPbI_3_ QDs, but they are still limited to achieving the synthesis of pure red QDs^24–26^.

In addition, the purification of QDs with polar antisolvent magnifies the proton transfer process between OA^-^ (deprotonated OA) and OAmH^+^ (protonated OAm) ligands. It leads to the shedding of the ligands on the surface of QDs and the formation of non-radiative recombination traps, which deteriorates the stability and optical properties of QDs^27,28^.

**Fig. 1:**
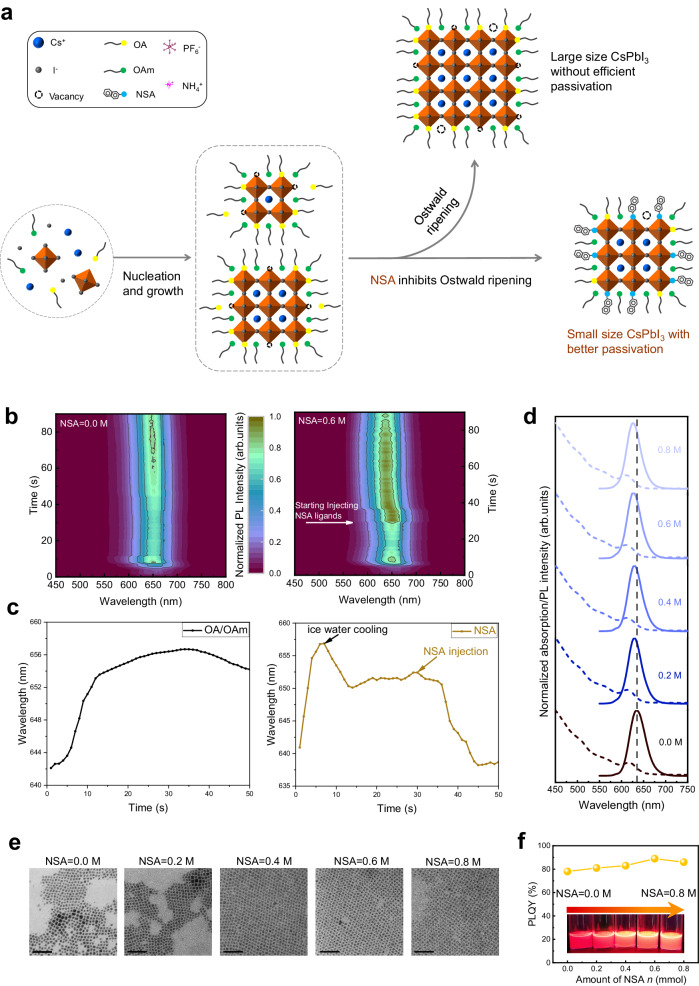
Nucleation and growth of CsPbI3 QDs with different ligands. **a** Schematic diagram of 2-naphthalenesulfonic acid (NSA) ligand passivating CsPbI_3_ QDs and inhibiting Ostwald ripening. **b** In-situ photoluminescence (PL) spectra of CsPbI_3_ QDs with or without NSA treatment. c Wavelength evolution extracted from in-situ PL spectra of CsPbI_3_ QDs with or without NSA treatment. **d** Absorption and PL spectra of CsPbI_3_ QDs synthesized with different amounts of NSA (0.0,0.2,0.4,0.6,0.8 mmol (M)). **e** The TEM images (scale bar 50 nm) of CsPbI_3_ QDs synthesized with different NSA amounts and the corresponding size statistical distribution maps. **f** Photoluminescence Quantum Yield (PLQY) of CsPbI_3_ QDs synthesized with different NSA amounts. Inset: Photos of CsPbI_3_ QDs synthesized with different NSA amounts under ultraviolet light (365 nm).

Here, we succeed in synthesizing strong-confined CsPbI_3_ QDs with a pure red emission peak at 623 nm. We introduce 2-naphthalene sulfonic acid (NSA) to regulate the Ostwald ripening growth of QDs after nucleation. The injected NSA ligand can push the proton transfer process between OA^−^ and OAmH^+^, thus producing lots of OAm and removing OA/OAm ligands from QD surfaces. It is demonstrated here that the sulfonic acid group has stronger binding energy with Pb atoms on the QDs surface compared to OAm by DFT calculation, which replaces the original weak-bounding OAm ligands on the surface of QDs to reduce the generation of active perovskite ionic sites. The naphthalene ring of the NSA ligand also has a large steric hindrance, inhibiting the overgrowth of QDs. The schematical diagram of synthesizing NSA-treated CsPbI_3_ QDs is shown in Fig. 1a. In the purification process of QDs, ammonium hexafluorophosphate (NH_4_PF_6_) is introduced to strongly bind the QDs surface and passivate the defects, avoiding deteriorating the stability and optical properties of QDs in the purification process. Moreover, inorganic ligands can also improve the electrical conductivity of perovskite QDs. With the design of suppression overgrowth of QDs, monodispersed, strong-confined, and single-phase CsPbI_3_ QDs are synthesized, with an average size of about 4.3 nm and bright photoluminescence (PL) emission at 623 nm with full width at half maximum (FWHM) of 32 nm and PLQY of 94%. Pure red PeLED is fabricated based on strong quantum confinement CsPbI_3_ QDs, showing a maximum EQE of 26.04% for electroluminescence (EL) at 628 nm. The maximum luminance of our device is 4203 cd m^−2^ at 5.8 V, and the operational half-lifetime (*T*_50_) is 729 min at 1000 cd m^−2^.

## Results

### Inhibition of Ostwald ripening by strong ligands

In-situ PL spectroscopy studies the PL evolution of QDs nucleation and growth (Fig. 1b). The extracted wavelength evolution of in-situ PL spectra is manifested in Fig. 1c. After the injection of NSA ligand, in contrast with OA/OAm QDs (without NSA treatment), the blue shift of PL spectra and the enhancement of PL intensity of NSA-treated QDs denote that the harmful ripening is inhibited, and the defects are passivated. In contrast with OA, NSA has a higher dissociation constant and is more polar which can substitute the weak acid. NSA can promote the proton process between OA/OAm:

1 $${{{{{{\rm{OA}}}}}}}^{-}+{{{{{{\rm{OAmH}}}}}}}^{+}\to {{{{{\rm{OA}}}}}}+{{{{{\rm{OAm}}}}}}$$

Thus, NSA can facilitate debonding surface OA/OAm ligands and etch defective octahedra.

The PL and absorption spectra of QDs implanted with different amounts of NSA (0.2, 0.4, 0.6, 0.8 M) are shown in Fig. 1d to optimize the concentration of injected NSA ligands. The QDs synthesized without NSA treatment have an emission peak at 635 nm with an FWHM of 41 nm. With the increase in the amount of NSA, the PL peak of QDs blue shifts from 630 nm (0.2 M) to 626 nm (0.6 M). There is no significant change in the PL emission peak when the amount of NSA increases from 0.6 M to 0.8 M. Figure 1e is the TEM image and corresponding size statistics of QDs synthesized with the different amounts of injected NSA ligands. The particle size decreases with the increasing amount of NSA and the size distribution is also narrowed. The smaller and narrowed size distribution of NSA-treated QDs indicates that Ostwald ripening is prohibited.

**Fig. 2:**
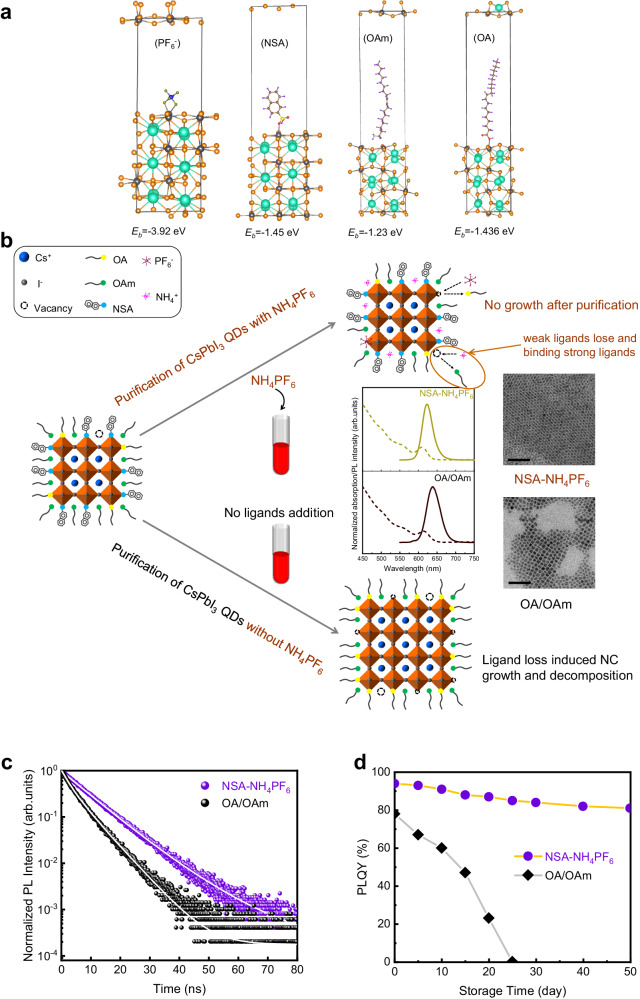
Binding energy of different ligands and properties of synthesized CsPbI3 QDs. **a** Calculated binding energy of oleic acid (OA), oleylamine (OA), 2-naphthalenesulfonic acid (NSA) and hexafluorophosphate (NH_4_PF_6_) ligands through DFT (**b**) schematic diagram of NH_4_PF_6_ ligand exchange process during purification including comparison of TEM image (scale bar 50 nm), absorption spectra and photoluminescence (PL) spectra of different QDs (**c**) Time-resolved photoluminescence (TRPL) decay of OA/OAm QDs and NSA-NH_4_PF_6_ QDs films. **d** Photoluminescence quantum yield (PLQY) variation of QDs solution after different storage times.

The injection of NSA ligands also increases the PLQY of QDs (as shown in Fig. 1f). When the amount of NSA is 0.6 M, PLQY reaches the highest value of 89%. The increased PLQY means that NSA ligands can passivate the QDs. The DFT calculated NSA binding energy is 1.45 eV which has stronger binding energy than OAm whose binding energy is only 1.23 eV as Fig. 2a exhibits. The stronger binding indicates that NSA can improve QD surface stability. The stronger binding of NSA is also demonstrated experimentally. The measured Fourier-transform infrared spectroscopy (FTIR) in Supplementary Fig. 2a implies there are NSA ligands that bind to QDs. The X-ray photoelectron spectroscopy (XPS) characterization in Supplementary Fig. 2b shows that the binding energy of Pb *4* *f* shifts to a higher energy side, indicating NSA has a stronger binding interaction with Pb atoms on the surface of QDs. ^1^H-NMR spectra of NSA-treated QDs (Supplementary Fig. 3) further ensure that NSA ligands are binding to QDs, which coincides with FTIR results. After the OA/OAm QDs (without NSA treatment) are stored for 3 days, the non-perovskite yellow phase and the fusion growth of QDs appear (Supplementary Fig. 4a). The corresponding TEM image (Supplementary Fig. 4b) shows that the QDs are fused into large-size nanocrystals, which may be the self-assembly of QDs caused by the debonding of weakly bound ligands^29^. However, the QDs treated with NSA (0.6 M) ligands maintain good dispersion (Supplementary Fig. 4c). In summary, the introduction of NSA ligands inhibits the ripening of QDs in the synthesis process, improving the optical properties and stability of QDs.

### Monodisperse strong-confined nanocrystals

After the NSA ligand treatment, the weak binding ligand on the QDs surface is further exchanged with the strong binding NH_4_PF_6_ ligand in the purification process to improve the photophysical properties of the CsPbI_3_ QDs. The DFT calculated PF_6_ anions binding energy is 3.92 eV, which is much higher than OA/OAm. We characterize the morphology and photophysical properties of QDs obtained with or without NH_4_PF_6_ ligand treatments. Figure 2b shows the absorption and PL spectra of the purified CsPbI_3_ QDs. The PL peak of CsPbI_3_ QDs treated with NSA and NH_4_PF_6_ ligands is 623 nm with an FWHM of 32 nm, while the PL peak of untreated CsPbI_3_ QDs is 639 nm with an FWHM of 42 nm. The measured PLQY of NSA-NH_4_PF_6_ CsPbI_3_ QDs is 94%, indicating that NH_4_PF_6_ ligands further passivated the surface defects. Compared to organic ligands, inorganic ligands achieve higher ligands density and passivate deep defects due to lower steric hindrance.

TEM images of CsPbI_3_ QDs with OA/OAm and NSA-NH_4_PF_6_ are shown in Fig. 2b. Their size distribution statistics comparison is manifested in Supplementary Fig. 5, from which the NSA-NH_4_PF_6_ QDs exhibit significantly smaller sizes and narrower size distribution than OA/OAm QDs. Generally, the polar solvent removes OA/OAm ligands during purification and exposes the surface defects. However, the strong ligands inhibit the NSA-NH_4_PF_6_ QDs regrowth or decomposition in the purification process as NH_4_PF_6_ immediately passivates the surface after OA/OAm is removed.

Time-resolved photoluminescence (TRPL) decay spectra analysis is shown in Fig. 2c. The average PL lifetime of NSA-NH_4_PF_6_ QDs film is 7.9 ns, longer than that of OA/OAm QDs film (4.3 ns). The result represents that the NSA-NH_4_PF_6_ QDs film non-radiative recombination is suppressed and increases the PLQY of QDs. The synthesized CsPbI_3_ QDs are aged in the air. After 25 days, the OA/OAm QDs decomposed and the PLQY decreased to 0, while NSA-NH_4_PF_6_ QDs maintained 80% of the initial PLQY after 50 days of storage (Fig. 2d). The enhanced stability is attributed to the strong binding of NH_4_PF_6_ ligands. Our strategy is demonstrated to successfully synthesize pure red CsPbI_3_ QDs with excellent optical properties and stabilities.

### Strong binding of ligands on nanocrystals

**Fig. 3:**
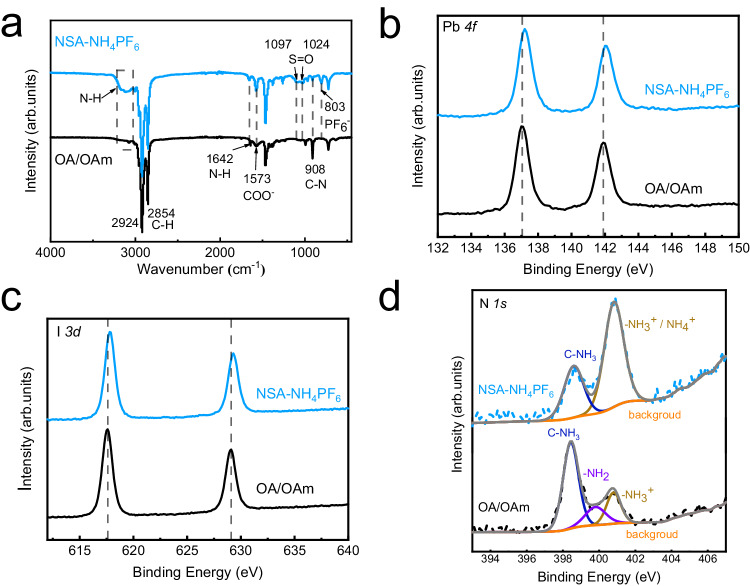
Chemical binding characterizations of different ligands synthesized CsPbI3 QDs. **a** Fourier transform infrared spectroscopy (FTIR) of oleic acid/oleylamine (OA/OAm) and 2-naphthalenesulfonic acid hexafluorophosphate (NSA-NH_4_PF_6_) QDs; for OA/OAm QDs, the peaks at 1750 cm^−1^ and 1573 cm^−1^ are attributed to the stretching vibration of C=O and oleate anion (-COO^−^) of OA, respectively, and the signal peaks at 1642 cm^−1^ and 908 cm^−1^ can be attributed to N-H bending vibration and C-N stretching vibration of OAm, respectively^34,35^. X-ray photoelectron spectroscopy (XPS) of (**b**) Pb *4* *f*, (**c**) I *3d*, and (**d**) N *1* *s* of OA/OAm QDs and NSA-NH_4_PF_6_ QDs.

To experimentally demonstrate the strong binding of NSA and NH_4_PF_6_ ligands, we characterize the surface ligand states of NSA-NH_4_PF_6_ QDs and OA/OAm QDs. Figure 3a shows the results of FTIR. After introducing the NSA and NH_4_PF_6_ ligands in the synthesis and purification process respectively, there are two peaks at 1097 cm^−1^ and 1024 cm^−1^ for NSA-NH_4_PF_6_ QDs which can be attributed to the stretching vibration of the sulfonyl group^30,31^, and the signal of stretching vibration of the PF_6_ group is also detected at 803 cm^−1^^32^. In addition, for NSA-NH_4_PF_6_ QDs, an N-H tensile signal corresponding to the ${{{{{{\rm{NH}}}}}}}_{4}^{+}$ is presented^32^, indicating that ${{{{{{\rm{NH}}}}}}}_{4}^{+}$ of NH_4_PF_6_ bonding to QDs. The intensity of the characteristic peak of OAm at 908 cm^−1^ (C-N) decreases significantly, indicating the successful exchange of OAm ligands. These results indicate the ligand exchange on the surface of QDs. The substantial weakly bounded OA/OAm ligands are replaced by NSA and NH_4_PF_6_ ligands in the process of synthesis and purification of QDs.

The chemical states of the surface elements of CsPbI_3_ QDs synthesized by different ligands are further characterized by XPS. Figure 3b are Pb *4* *f* spectra of synthesized QD films, while Fig. 3c are I *3d* spectra. Compared to OA/OAm QDs, the binding energies of Pb *4* *f* and I *3d* of NSA-NH_4_PF_6_ QDs move to a higher level, which suggests that NSA and PF_6_ anions bind to Pb more strongly than OA. Furthermore, from the XPS spectrum of N *1* *s* in Fig. 3d, compared to OA/OAm QDs, the peak of C-NH_3_, belonging to OAm, is weakened and the peak belonging to ${-{{{{{\rm{NH}}}}}}}_{3}^{+}$ and ${{{{{{\rm{NH}}}}}}}_{4}^{+}$ is enhanced after treatment with NSA and NH_4_PF_6_ ligands. In addition to the disappearance of the amine group (-NH_2_) at 399.8 eV, we believe that NSA replaces OAm, and ${{{{{{\rm{NH}}}}}}}_{4}^{+}$ replaces OAmH^+^.

### Efficient and stable devices

CsPbI_3_ QDs treated with different ligands are employed as emitting layers to fabricate QLEDs. The root-mean-square (RMS) roughness of NSA-NH_4_PF_6_ CsPbI_3_ QD films is only 1.33 nm (Supplementary Fig. 8a), which is lower than that of OA-OAm QD films (RMS = 5.18 nm) (Supplementary Fig. 8b). Supplementary Fig. 9 shows the structure and thickness of QLEDs, which is ITO/ PEDOT: PSS/ PTAA/ CsPbI_3_ QDs/ TmPyPB/ PO-T2T/ LiF/ Al. The EQE limit of our device is explored by simulation (Supplementary Fig. 10). We systematically vary the thickness of the quantum dot emitting layer (Supplementary Fig. 11), electron transport layers (ETLs, Supplementary Figs. 12 and 13), and hole transport layer (HTL, Supplementary Fig. 14) to investigate their influence on the optical properties and mode distribution within the waveguide structure.

**Fig. 4:**
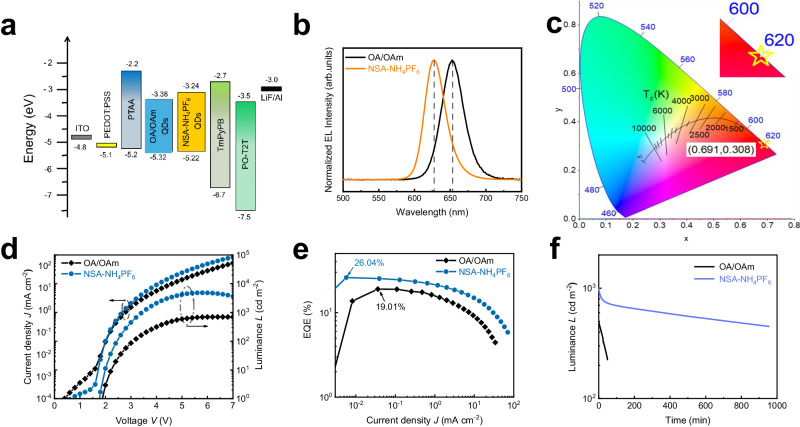
Device performance of different ligands synthesized CsPbI3 QDs. **a** The energy level diagram of light-emitting diodes (LED) devices. **b** The normalized electroluminescence (EL) spectrum of oleic acid/oleylamine (OA/OAm) and 2-naphthalenesulfonic acid hexafluorophosphate (NSA-NH_4_PF_6_) QDs based LEDs. **c** The emission chromaticity diagram of LEDs is based on NSA-NH_4_PF_6_ QDs, with CIE coordinates of (0.691, 0.308). **d** *J-V* and *L-V* curves of different QDs based LEDs. **e** external quantum efficiency (EQE) versus current density (*J*) curves of different QDs based LEDs, inset shows a photo of the device lit at the voltage corresponding to the maximum brightness. **f** The operational stability of different QDs based LEDs.

The energy levels of CsPbI_3_ QDs (Fig. 4a) are calculated from Tauc plots (Supplementary Fig. 15) and UPS (Supplementary Fig. 16). The QLEDs fabricated with NSA-NH_4_PF_6_ CsPbI_3_ QDs show EL emission centered on 628 nm, with an FWHM of 33 nm (Fig. 4b), which have a pure red CIE coordinate of (0.691, 0.308) as shown in Fig. 4c. Figure 4d is the current density-voltage-luminance (*J-V-L*) curve of QLED devices. QLED devices based on NSA-NH_4_PF_6_ QDs possess lower turn-on voltage and higher current density, which indicates a more effective charge injection of NSA-NH_4_PF_6_ QDs. This can be attributed to the replacement of long-chain organic ligands by inorganic ligand NH_4_PF_6_, which improves the carrier transport ability of QDs. Figure 4e is the EQE-*J* curve of NSA-NH_4_PF_6_ QDs-based QLEDs and controlled QLEDs (OA/OAm QDs-based devices). According to Fig. 4d, e, the NSA-NH_4_PF_6_ QDs-based QLEDs achieved the maximum luminance of 4203 cd m^−2^ at 5.8 V with a peak EQE of 26.04%. Meanwhile, the EQE of controlled devices is 19.01% with a maximum luminance of 696 cd m^−2^. The EQE distribution obtained from 25 devices manifests great reproducibility (Supplementary Fig. 17). We evaluate the operational stability of QLEDs (Fig. 4f). For the device prepared by NSA-NH_4_PF_6_ CsPbI_3_ QDs, the half-lifetime (*T*_50_, defined as the time when the brightness drops to half of the initial brightness) of devices is about 729 min at the initial brightness of 1000 cd m^−2^, while *T*_50_ of devices based on OA/OAm CsPbI_3_ QDs is about 43 min (*L*_0_ = 500 cd m^−2^). The EL spectra of NSA-NH_4_PF_6_ QDs-based devices under different applied voltages are the same (Supplementary Fig. 18). The performance of our QLEDs device represents the recording efficiency and lifetime of QLEDs based on perovskite colloid QDs in the visible region (Supplementary Table 1). Through this strategy, the efficient pure red emission of QLEDs based on CsPbI_3_ QDs is realized, which exhibits the state-of-the-art performance of pure red PeLEDs (Supplementary Table 2).

## Discussion

In conclusion, we have developed an effective strategy to realize stable and efficient strong confined pure phase perovskite QDs. By introducing a strong binding ligand NSA with large steric hindrance in the reaction quenching stage of QDs synthesis, the ripening and growth of CsPbI_3_ QDs are inhibited. The presence of the inorganic ligand NH_4_PF_6_ to exchange dynamically combined OA and OAm ligands passivate defects and improve the conductivity of QDs. Through this strategy, we synthesize small-size monodispersed CsPbI_3_ QDs (4.3 ± 0.6 nm) with a PLQY of 94%. The PL peak is 623 nm with an FWHM of 32 nm. Furthermore, we fabricate pure red (EL peak of 628 nm) PeLEDs with a maximum EQE of 26.04%. This strategy provides a way to solve the ripening and growth of perovskite QDs in the synthesis process, paving the way for full-color displays, which have a broad application prospect in optoelectronic devices.

## Methods

### Chemicals

Lead iodide (PbI_2_, Macklin, 99.999%), zinc iodide (ZnI_2_, Aladdin, 99.999%), cesium carbonate (Cs_2_CO_3_, Sigma-Aldrich), Octadecene (ODE, Sigma-Aldrich, 90%), oleic acid (OA, Sigma-Aldrich, 90%), oleylamine (OAm, Aladdin, 90%), n-hexane (Aladdin, 98%), n-octane (Aladdin, 99%), methyl acetate (Sinopharm Chemical Reagent Co., Ltd. (SCRC), 98%), ethyl acetate (Aladdin, 99.9%), 2-naphthalenesulfonic acid (NSA, SCRC, 98%), ammonium hexafluorophosphate (NH_4_PF_6_, Sigma-Aldrich, 99.999%), poly(3,4-ethylenedioxythiophene) polystyrene sulfonate (PEDOT: PSS, 4083, Xi’an Polymer Light Technology Corp.), poly[bis(4-phenyl)(2,4,6-trimethylphenyl)amine](PTAA, Lingzhi Technology Co., Ltd.), 2,4,6-tris[3-(diphenylphosphinyl)phenyl]−1,3,5-triazine (PO-T2T, Xi’an Polymer Light Technology Corp.), 3,3′-[5′-[3-(3-pyridinyl)phenyl][1,1’:3′,1”-terphenyl]−3,3”-diyl]bispyridine (TmPyPB, Xi’an Polymer Light Technology Corp.), lithium fluoride (LiF, Xi’an Polymer Light Technology Corp., 99.9%), which were directly used.

### Preparation of 2-naphthalenesulfonic acid ligand solution

0.2, 0.4, 0.6, and 0.8 mmol(M) NSA ligands were respectively dissolved in 1 mL of ethyl acetate solvent, and heated and stirred at 80 °C for 20 min for subsequent ligand treatment.

### Preparation of ammonium hexafluorophosphate ligand solution

0.2 mmol NH_4_PF_6_ was dissolved in 1 mL methyl acetate solvent for subsequent ligand exchange.

### Preparation of Cesium Oleic Solution

Cs_2_CO_3_ (0.142 g) was loaded into a 50 mL 3-neck flask together with octadecene (11 mL, ODE) and oleic acid (0.6 mL), and the mixture was dried for 20 min at 40 °C under argon. The mixture was heated to 100 °C in a vacuum until the Cs_2_CO_3_ powder reacted with OA to form a transparent solution, and then the mixture was heated at 100 °C for 20 min under argon flow. The obtained Cs-OA solution was stored in an argon atmosphere and heated to 100 °C before use.

### Synthesis and purification of oleic/oleylamine nanocrystals

In brief, 0.185 g PbI_2_, 0.351 g ZnI_2_, and 6.0 mL ODE were dried in a 50 mL three-neck flask under argon flow for 20 min. The temperature was raised to 120 °C and dried in argon flow for 1 h, then 1.2 mL OA and 2 mL OAm were injected. When the solute in the mixed solution is completely dissolved, raise the temperature to 150 °C. Quickly inject 2.4 mL Cs-OA (solution prepared as described above). After 5 s, the reaction mixture was immediately cooled to 50 °C by immersing it in an ice-water bath.

The mixture was transferred into a 50 mL centrifuge tube and centrifuged at 1479 × *g* (where g is the earth’s gravity force) for 1 min. The unreacted precursor precipitation was removed and the upper solution was uniformly transferred to the two 50 mL centrifuge tubes. Ethyl acetate and methyl acetate were added to the crude solution of QDs (the volume ratio of QDs, ethyl acetate, and methyl acetate was 1:2:3) and centrifuged at 4531 × *g* for 2 min. The precipitate containing CsPbI_3_ QDs was redispersed in 1 mL n-hexane, centrifuged with 1479 × *g* for 1 min, and the non-perovskite precipitate was removed. Add 1 mL of the preliminarily purified QD solution into 6 mL of methyl acetate and 6 mL of ethyl acetate to precipitate again, and centrifuge at 4531 × *g* for 1 min. The obtained CsPbI_3_ QDs were redispersed in 0.5 mL octane and centrifuged at 1479 × *g* for 1 min to remove aggregates and non-perovskite particles. Finally, the QD solution was filtered with a 0.22 μm nylon filter. The synthesized QDs are stored in the refrigerator for further characterization and device preparation. The QDs synthesized with conventional OA and OAm ligands are defined as OA/OAm CsPbI_3_ QDs.

### Synthesis and purification of strong ligand nanocrystals

In brief, 0.185 g PbI_2_, 0.351 g ZnI_2_, and 6.0 mL ODE were dried in a 50 mL three-neck flask under argon flow for 20 min. The temperature was raised to 120 °C and dried in argon flow for 1 h, then 1.2 mL OA and 2 mL OAm were injected. When the solute in the mixed solution is completely dissolved, raise the temperature to 150 °C. Quickly inject 2.4 mL Cs-OA (solution prepared as described above). After 5 s, the reaction mixture was immersed in a cold water bath. When the temperature cooled to 120 °C, the NSA ligand solution was slowly injected (about 5 s), and then the reaction mixture was cooled to 50 °C in an ice-water bath.

The first purification process of crude solution is the same as that of OA/OAm CsPbI_3_ QDs mentioned above. The QD solution (1 mL) was added to the mixed solution of 6 mL ethyl acetate and 120 μL NH_4_PF_6_ ligand solution and oscillated for 10 s. Centrifuge at 2311 × *g* for 1 min to remove the precipitation of aggregates and non-perovskite particles. 6 mL of methyl acetate was added to the upper solution and centrifuged at 4531 × *g* for 1 min to obtain a precipitate containing CsPbI_3_ QDs. The obtained CsPbI_3_ QDs were redispersed in 0.5 mL octane and the impurities were removed by centrifugation for 1 min at 1479 × *g*. Finally, the QD solution was filtered with a 0.22 μm nylon filter. The synthesized QDs are stored in the refrigerator for further characterization and device preparation. The QDs treated with NSA and NH_4_PF_6_ ligands are defined as NSA-NH_4_PF_6_ CsPbI_3_ QDs.

### Fabrication and characterization of devices

Patterned ITO-coated glasses were ultrasonically cleaned with an ITO cleaning solution, deionized water, acetone, isopropanol, and ethanol for 30 min each. Then, the ITO-coated glasses were dried with dry N_2_ and placed into a UV-ozone cleaner for 30 min. The PEDOT: PSS was spin-coated onto an ITO-coated glass at 5500 rpm for 50 s and annealed at 150 °C for 20 min in the air. The coated substrate was moved into a nitrogen glove box. A solution of PTAA in chlorobenzene (8 mg mL^−1^) was spin-coated on the PEDOT: PSS layer by the spin-coater at 1500 rpm for 45 s and annealed at 120 °C for 20 min under a nitrogen atmosphere. The CsPbI_3_ QDs in octane were spin-coated onto the PTAA layer by the spin-coater at 4000 rpm for 45 s and annealed at 60 °C for 5 min. Afterward, 6 nm TmPyPB layer, 37 nm of PO-T2T layer, 1 nm of LiF layer, and 60 nm of Al electrode were deposited by a thermal evaporation system equipped with a shadow mask under a high vacuum (2 × 10^−4^ Pa). The device’s active area was 4 mm^−2^. For the as-fabricated LED devices performance tests, the electroluminescence performance of devices was measured with a setup described following^33^. We used an Ocean Optics USB 2000+ spectrometer to gain the electroluminescence spectra. The devices were driven at a constant current with a Keithley 2400 source meter. The *J*-*L*-*V* characteristics of the devices were measured in ambient conditions. The Keithley 2400 source meter was used to monitor the sweeping voltages and currents. Additionally, light intensity was measured by a Keithley 6485 Picoammeter integrated with a calibrated silicon detector (Edmund). Luminance was calibrated using a luminance meter (Konica Minolta LS-160) with the assumption that all devices are Lambertian emission patterns. The operation lifetimes of devices were measured under ambient conditions using a commercialized lifetime test system (Guangzhou Jinghe Equipment Co., Ltd.).

### Characterizations

The ultraviolet-visible (UV-vis) absorption spectrum of the QD solution was measured by a PerkinElmer instrument. PL spectra of the QD solution were obtained by using a HORIBA fluorescence spectrometer (FL3-111). The PLQY value of QDs diluted with hexane was measured by the Otsuka QE2100 device. Transmission electron microscopy (TEM) images and high-angle annular dark-field scanning transmission electron microscopy (HAADF-STEM) images were obtained by Talos F200X instrument, and the accelerating voltage was 200 kV. The energy-dispersive X-ray spectroscopy (EDS) spectra were collected on a Talos F200X instrument with an energy-dispersive detector. Atomic force microscope (AFM) images of QD films were measured by Dimension ICON instrument. The X-ray diffraction (XRD) pattern of the QD powder was recorded using a D8 ADVANCE diffractometer with Cu Kα radiation (λ = 1.54178 Å). The FTIR spectrum of QD surface ligands was measured by Fourier infrared spectrometer (IS50). Time-resolved PL decay spectra of QDs were obtained by an FL3-111 spectrometer coupled with a 457 nm, 45 ps pulsed laser, and a time-corrected single-photon counting system.

### Simulation of device optical waveguide mode distribution

The optical waveguide mode distribution was simulated using the Setfos 5.4 software (Serial Number: 1127696771). The device structure, as depicted in Supplementary Fig. 10, served as the basis for the simulations. We systematically varied the thickness of the quantum dot emitting layer, ETL, and HTL to investigate their influence on the optical properties and mode distribution within the waveguide structure. The simulated results were visualized and analyzed, with the corresponding mode distributions presented in Supplementary Figs. 10–14.

### Supplementary information

Supplementary Information

Peer Review File

## Data Availability

The data supporting the findings of this study are available in the paper, Supplementary Information, as well as from the corresponding authors upon request.

## References

[1] Shamsi J, Urban AS, Imran M, De Trizio L, Manna L (2019). Metal halide perovskite nanocrystals: synthesis, post-synthesis modifications, and their optical properties. Chem. Rev..

[2] Li Y, Zhang X, Huang H, Kershaw SV, Rogach AL (2020). Advances in metal halide perovskite nanocrystals: synthetic strategies, growth mechanisms, and optoelectronic applications. Mater. Today.

[3] Protesescu L (2015). Nanocrystals of cesium lead halide perovskites (CsPbX3, X= Cl, Br, and I): novel optoelectronic materials showing bright emission with wide color gamut. Nano Lett..

[4] Manser JS, Christians JA, Kamat PV (2016). Intriguing optoelectronic properties of metal halide perovskites. Chem. Rev..

[5] Wan Q (2023). Ultrathin light-emitting diodes with external efficiency over 26% based on resurfaced Perovskite nanocrystals. ACS Energy Lett..

[6] Jiang J (2022). Red Perovskite light‐emitting diodes with efficiency exceeding 25% realized by co‐spacer cations. Adv. Mater..

[7] Gao Y (2022). High‐performance perovskite light‐emitting diodes enabled by passivating defect and constructing dual energy‐transfer pathway through functional perovskite nanocrystals. Adv. Mater..

[8] Kim JS (2022). Ultra-bright, efficient and stable perovskite light-emitting diodes. Nature.

[9] Triana MA, Hsiang E-L, Zhang C, Dong Y, Wu S-T (2022). Luminescent nanomaterials for energy-efficient display and healthcare. ACS Energy Lett..

[10] Mir WJ (2022). Lecithin capping ligands enable ultrastable perovskite-phase CsPbI3 quantum dots for Rec. 2020 bright-red light-emitting diodes. J. Am. Chem. Soc..

[11] Lan Y-F (2021). Spectrally stable and efficient pure red CsPbI3 quantum dot light-emitting diodes enabled by sequential ligand post-treatment strategy. Nano Lett..

[12] Zhang H (2019). Phase segregation due to ion migration in all-inorganic mixed-halide perovskite nanocrystals. Nat. Commun..

[13] Vashishtha P, Halpert JE (2017). Field-driven ion migration and color instability in red-emitting mixed halide perovskite nanocrystal light-emitting diodes. Chem. Mater..

[14] Zhang L (2021). High-performance quasi-2D perovskite light-emitting diodes: from materials to devices. Light Sci. Appl..

[15] Liu X-K (2021). Metal halide perovskites for light-emitting diodes. Nat. Mater..

[16] Swarnkar A (2016). Quantum dot–induced phase stabilization of α-CsPbI3 perovskite for high-efficiency photovoltaics. Science.

[17] Xu L, Yu SH, Yang Y, Liang HW (2018). Stability and reactivity: positive and negative aspects for nanoparticle processing. Chem. Rev..

[18] Gao Q (2022). Halide perovskite crystallization processes and methods in nanocrystals, single crystals, and thin films. Adv. Mater..

[19] De Roo, J. et al. Highly Dynamic Ligand Binding and Light Absorption Coefficient of Cesium Lead Bromide Perovskite Nanocrystals. *ACS Nano* **10**, 2071–2081 (2016). 10.1021/acsnano.5b06295 26786064

[20] Fiuza-Maneiro, N. et al. Ligand Chemistry of Inorganic Lead Halide Perovskite Nanocrystals. *ACS Energy Lett.* **8**, 1152−1191 (2023).

[21] Koolyk M, Amgar D, Aharon S, Etgar L (2016). Kinetics of cesium lead halide perovskite nanoparticle growth; focusing and de-focusing of size distribution. Nanoscale.

[22] Yao J-S (2019). Few-nanometer-sized α-CsPbI3 quantum dots enabled by strontium substitution and iodide passivation for efficient red-light emitting diodes. J. Am. Chem. Soc..

[23] Wang YK (2021). All‐inorganic quantum‐dot LEDs based on a phase‐stabilized Α‐CsPbI3 perovskite. Angew. Chem. Int. Ed..

[24] Li H (2023). In-situ reacted multiple-anchoring ligands to produce highly photo-thermal resistant CsPbI3 quantum dots for display backlights. Chem. Eng. J..

[25] Zhang J, Yin C, Yang F, Yao Y, Hou L (2021). Highly luminescent and stable CsPbI 3 perovskite nanocrystals with sodium dodecyl sulfate ligand passivation for red-light-emitting diodes. J. Phys. Chem. Lett..

[26] Pan J (2017). Bidentate ligand-passivated CsPbI3 perovskite nanocrystals for stable near-unity photoluminescence quantum yield and efficient red light-emitting diodes. J. Am. Chem. Soc..

[27] De Roo J (2016). Highly dynamic ligand binding and light absorption coefficient of cesium lead bromide perovskite nanocrystals. ACS Nano.

[28] Yang D (2019). CsPbBr3 quantum dots 2.0: benzenesulfonic acid equivalent ligand awakens complete purification. Adv. Mater..

[29] Sun JK (2018). Polar solvent induced lattice distortion of cubic CsPbI(3) nanocubes and hierarchical self-assembly into orthorhombic single-crystalline nanowires. J. Am. Chem. Soc..

[30] Qiu X (2021). Highly efficient re-cycle/generation of LiCoO2 cathode assisted by 2-naphthalenesulfonic acid. J. Hazard. Mater..

[31] Zhao H (2021). High-brightness perovskite light-emitting diodes based on FAPbBr3 nanocrystals with rationally designed aromatic ligands. ACS Energy Lett..

[32] Mishra K, Hashmi S, Rai D (2013). Investigations on poly (ethylene oxide)+ NH4PF6 solid polymer electrolyte system. Int. J. Polymeric Mater. Polymeric Biomater..

[33] Mashford BS (2013). High-efficiency quantum-dot light-emitting devices with enhanced charge injection. Nat. Photonics.

[34] Li X (2021). Heterogeneous post-passivation of inorganic cesium lead halide perovskite quantum dots for efficient electroluminescent devices. J. Mater. Chem. C.

[35] Bi C (2021). Perovskite quantum dots with ultralow trap density by acid etching‐driven ligand exchange for high luminance and stable pure‐blue light‐emitting diodes. Adv. Mater..

