# Supplementary material for: Stable and efficient CsPbI3 quantum-dot light-emitting diodes with strong quantum confinement
Source: Nat Commun. 2024 Jul 7;15:5696. doi: 10.1038/s41467-024-50022-8 (PMC11228028; doi:10.1038/s41467-024-50022-8)
Supplement: Supplementary file 1 — Supplementary Information [file 41467_2024_50022_MOESM1_ESM.pdf]

Supporting Information

Stable and Efficient $CsPbI_3$ Quantum-Dot Light-Emitting Diodes with Strong Quantum Confinement

Yanming Li[1,2‡], Ming Deng[1,2,3‡], Xuanyu Zhang[1,2,4‡], Ting Xu[5‡], Ximeng Wang[6], Zhiwei Yao[1,2], Qiangqiang Wang[1,2,3], Lei Qian[1,2,7], Chaoyu Xiang[1,2,7]*

Corresponding author: Chaoyu Xiang

*Email: xiangchaoyu@nimte.ac.cn

‡ These authors contributed equally.

1. Laboratory of Advanced Nano-Optoelectronic Materials and Devices, Ningbo Institute of Materials Technology and Engineering, Chinese Academy of Science, Ningbo, Zhejiang, 315201, China
2. Laboratory of Advanced Nano-Optoelectronic Materials and Devices, Qianwan Institute of CNITECH, Ningbo, P. R. China, Ningbo 315300, China
3. Ningbo University, Ningbo, Zhejiang 315211, China
4. University of Nottingham Ningbo China, Ningbo 315100, China
5. Institute of Information Technology, Shenzhen Institute of Information Technology, Shenzhen, China
6. Department of Engineering Physics, University of Wisconsin-Madison, Madison, WI, 53706, USA
7. Zhejiang Provincial Engineering Research Center of Energy Optoelectronic Materials and Devices, Ningbo Institute of Materials Technology & Engineering,

Chinese Academy of Sciences

Density Functional Theory Calculations

The binding energies between different ligands and $CsPbI_3$ are calculated using density functional theory (DFT) in the Vienna ab initio Simulation Package (VASP) [1,2]with projector augmented waves (PAW) pseudopotentials[3,4]. The generalized gradient approximation (GGA) is chosen to approximate the exchange-correlation energy with the Perdew–Burke–Ernzerhoff (PBE) functional[5]. We use a cutoff energy of 500 eV and an 8 × 8 × 1 Monkhorst–Pack k-point mesh for all the calculations[6]. The convergence threshold for each ionic step is set to be 1 × 10^{-6} eV and the convergence threshold of maximum force is set to be 0.025 eV $Å^{-1}$. Spin polarization is also included in our calculations. All the calculations are based on the Ferromagnetic (FM) configuration. We chose (100) surfaces to simulate the binding process as the high-resolution TEM image of QDs manifested. Both ion positions and cell shape are allowed to change during the structural relaxation. The configurations of ligand-$CsPbI_3$ binding are shown in Supplementary Figure 1 in supporting information. After structural relaxation, the binding energy is calculated by the equation:

$$E_{\mathrm{b}} = E_{\mathrm{ligand-CsPbI_3}} - E_{\mathrm{ligand}} - E_{\mathrm{CsPbI_3}} \quad (1).$$

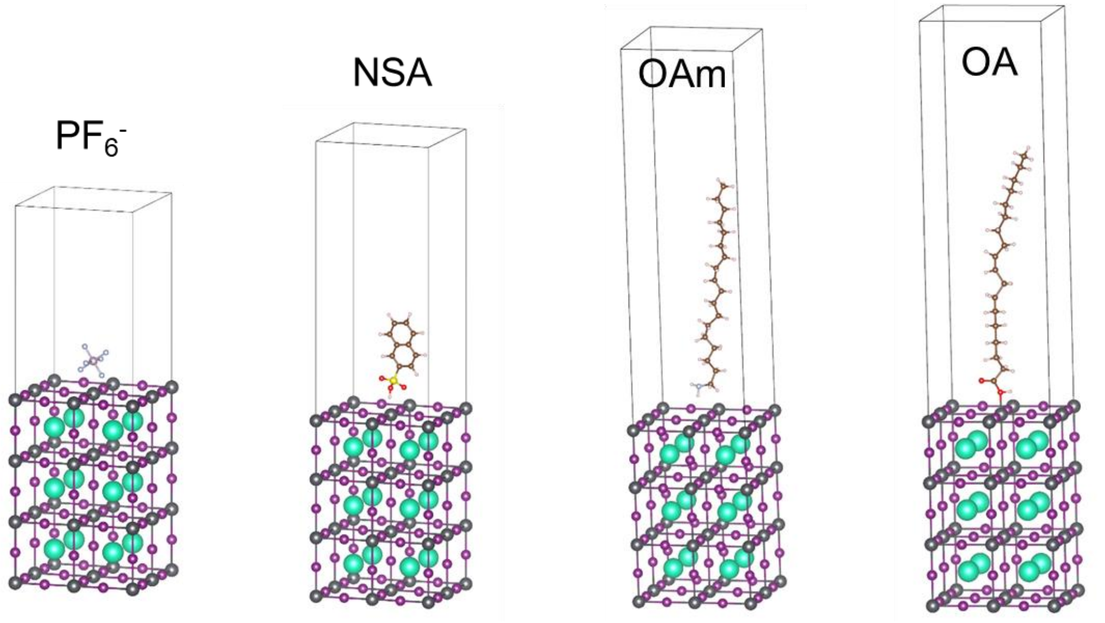

Supplementary Figure 1 Different ligands-$CsPbI_3$ Binding. Configurations of various ligand-$CsPbI_3$ binding including PF_6 anions, 2-naphthalenesulfonic acid (NSA), oleic acid (OA), oleylamine (OAm).

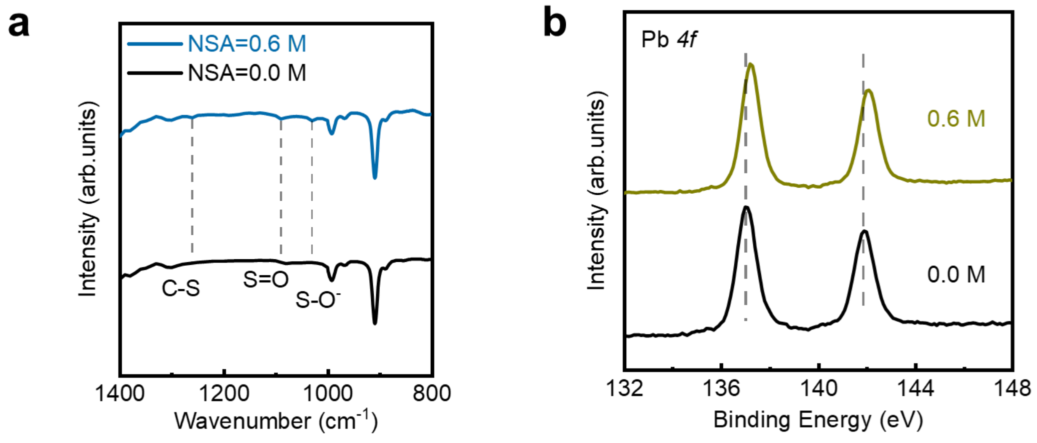

Supplementary Figure 2 Chemical binding characterization of QDs with or without 2-naphthalenesulfonic acid. (a) Fourier transform infrared spectroscopy (FTIR) characterization of $CsPbI_3$ QDs synthesized with 0 mmol (M) and 0.6 M of 2-naphthalenesulfonic acid (NSA); the stretching vibration of S-O^- and S=O

originates from the sulfonic acid group of the NSA ligand. (b) X-ray photoelectron spectroscopy (XPS) characterization of $CsPbI_3$ QDs with different NSA content; with 0 M NSA, Pb *4f* binding energy peaks are 137.0 eV and 141.9 eV; with 0.6 M, Pb *4f* binding energy peaks are 137.2 eV and 142.1 eV.

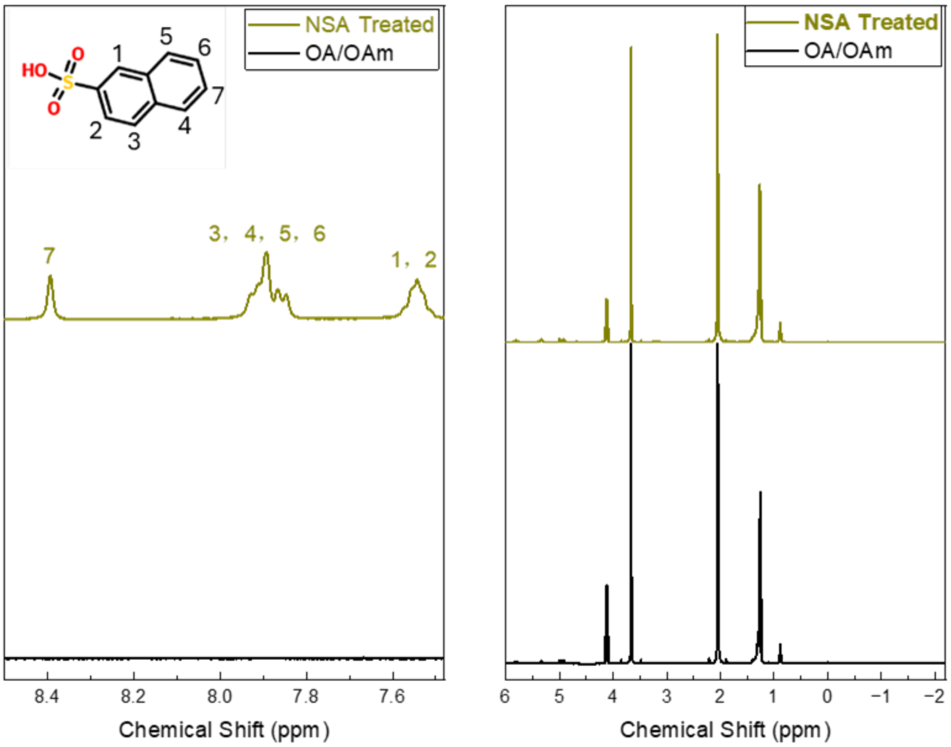

Supplementary Figure 3 ^{1}H-NMR spectra of QDs with or without 2-naphthalenesulfonic acid. ^{1}H-NMR spectra of 2-naphthalenesulfonic acid (NSA) treated nanocrystals and oleic acid/oleylamine (OA/OAm) nanocrystals without treatment.

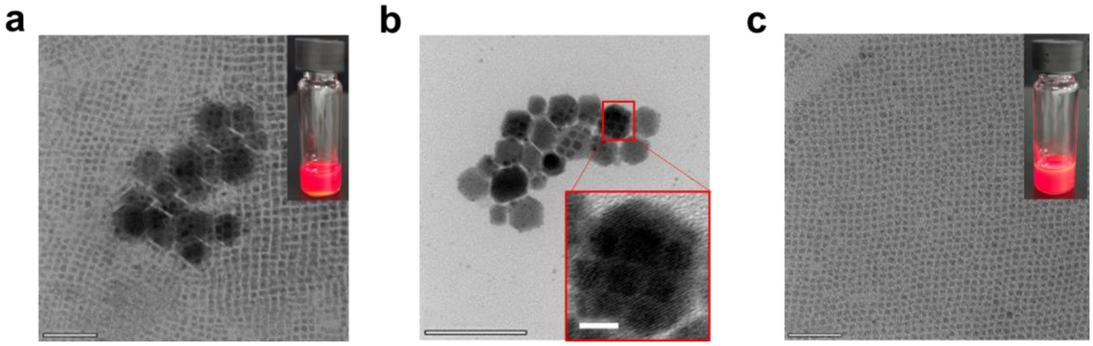

Supplementary Figure 4 Storage Stability of QDs with or without 2-naphthalenesulfonic acid. TEM images of $CsPbI_3$ QDs synthesized with different 2-naphthalenesulfonic acid (NSA) amounts at ambient storage (temperature 25±5 °C, humidity 50±10%) for 3 days, the illustration is a photograph of the corresponding QDs in natural light, (a) NSA (0.0 mmol, scale bar 50 nm). (b) The TEM image of $CsPbI_3$ QDs with NSA of 0.0 mmol (scale bar 100 nm); the QDs are fused into large-size nanocrystals, and the illustrations are high-resolution TEM images of nanocrystals (scale bar 10 nm). (c) NSA (0.6 mmol, scale bar 50 nm).

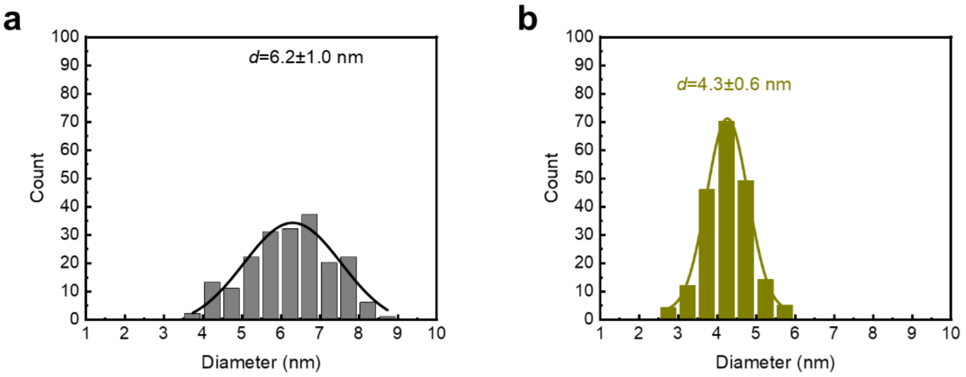

Supplementary Figure 5 Size distribution of different QDs. QDs size statistics corresponding to TEM images of (a) oleic acid/ oleylamine (OA/OAm) QDs and (b) 2-naphthalenesulfonic acid hexafluorophosphate (NSA-NH_4PF_6) QDs.

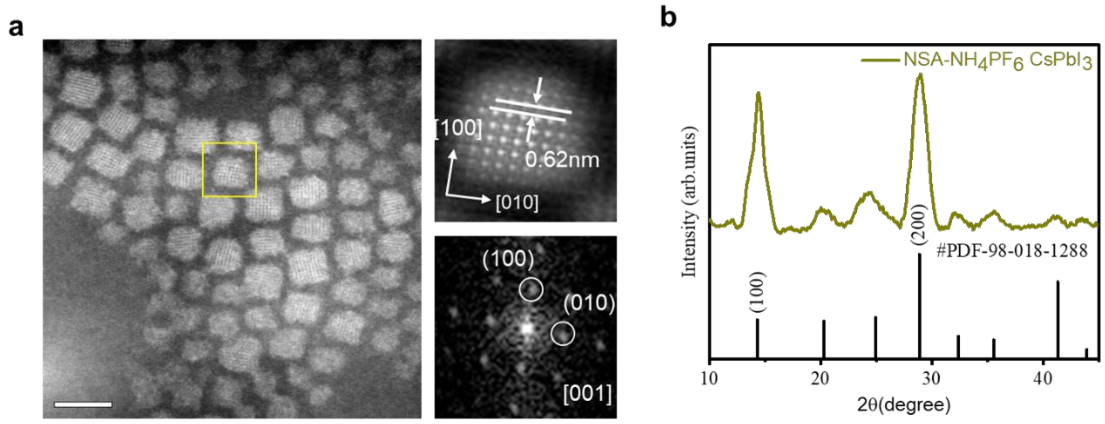

Supplementary Figure 6 Crystallographic information of 2-naphthalenesulfonic acid hexafluorophosphate QDs. (a) High-resolution TEM images (scale bar 10 nm) of 2-naphthalenesulfonic acid hexafluorophosphate (NSA-NH_4PF_6) $CsPbI_3$ QDs (b) X-ray diffraction spectra (XRD) of NSA-NH_4PF_6 $CsPbI_3$ QD film and corresponding standard PDF card.

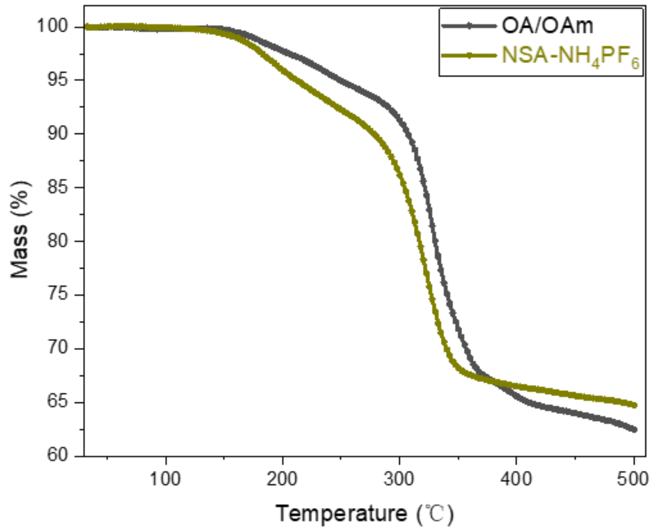

Supplementary Figure 7 Ligands content of different QDs. Thermo-gravimetric analysis (TGA) of 2-naphthalenesulfonic acid hexafluorophosphate (NSA-NH_4PF_6) QDs and oleic acid/oleylamine (OA/OAm) QDs.

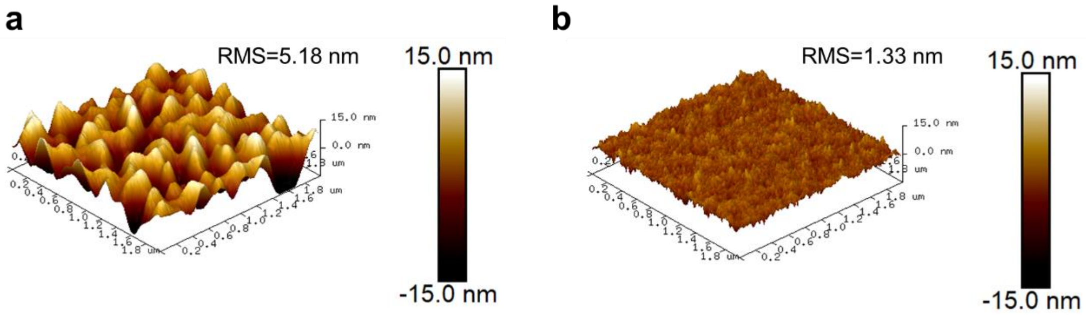

Supplementary Figure 8 Roughness of different QDs films. Atomic force microscope (AFM) images of $CsPbI_3$ QDs (a) oleic acid/oleylamine (OA/OAm) QDs, (b) 2-naphthalenesulfonic acid hexafluorophosphate (NSA-NH_4PF_6) QDs.

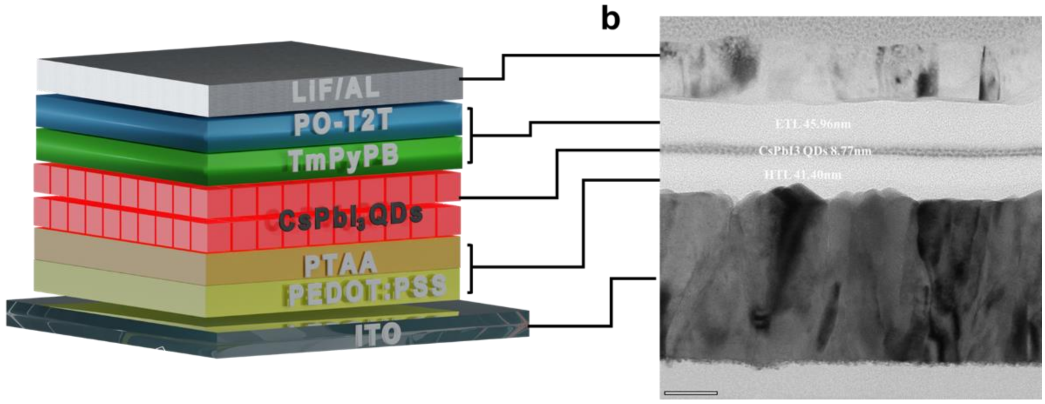

Supplementary Figure 9 Device structure and layer thickness. (a) $CsPbI_3$ quantum dot light-emitting diodes (QLEDs) device structure diagram. (b) Cross-sectional TEM images (scale bar 50 nm) of LEDs based on 2-naphthalenesulfonic acid hexafluorophosphate (NSA-NH_4PF_6) $CsPbI_3$ QDs.

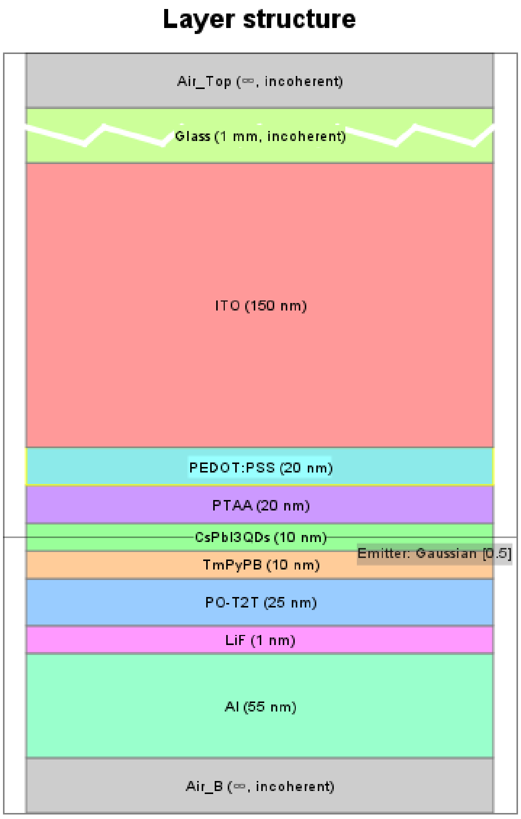

Supplementary Figure 10 Device structure for simulation. Device structure used for simulation of optical waveguide mode distribution.

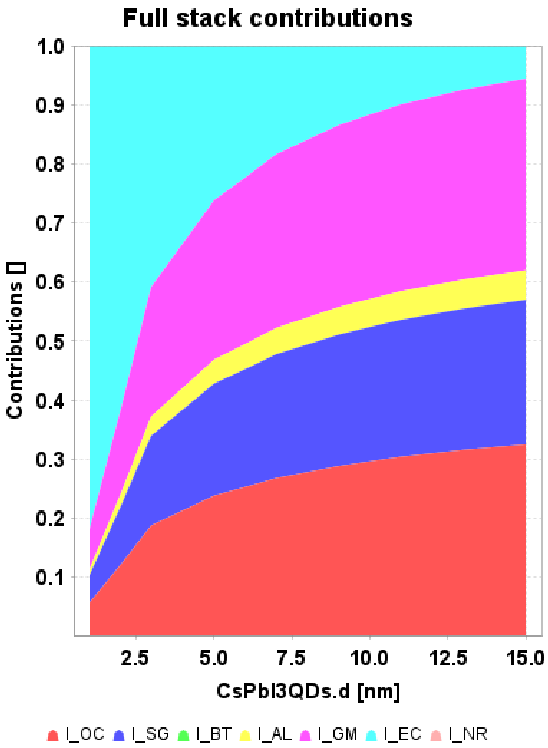

Supplementary Figure 11 Mode distribution influenced by QDs thickness. The mode distribution varies with the thickness of the quantum dot emitting layer within the waveguide structure.

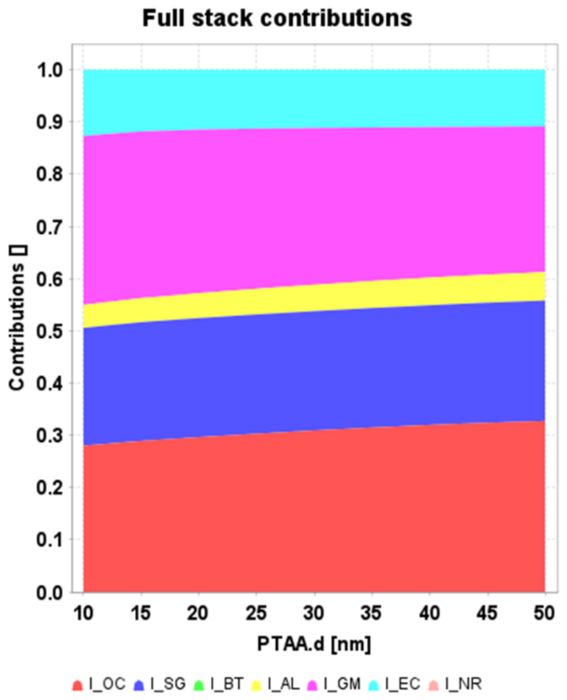

Supplementary Figure 12 Mode distribution influenced by PTAA thickness. The mode distribution varies with the thickness of the PTAA within the waveguide structure.

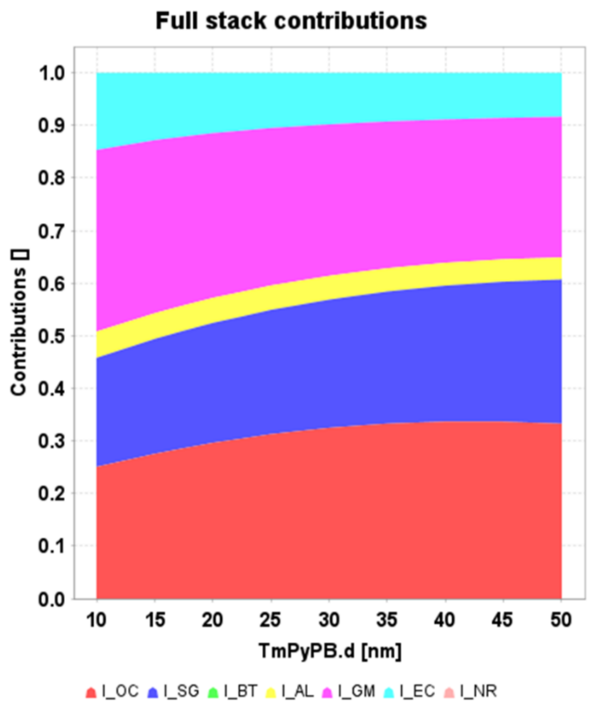

Supplementary Figure 13 Mode distribution influenced by TmPyPB thickness. The mode distribution varies with the thickness of the TmPyPB within the waveguide structure.

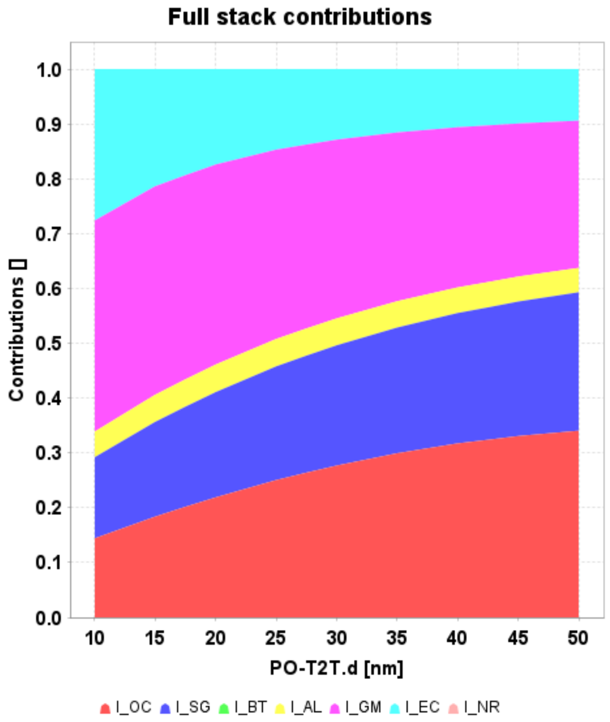

Supplementary Figure 14 Mode distribution influenced by PO-T2T thickness.

The mode distribution varies with the thickness of the PO-T2T within the waveguide structure.

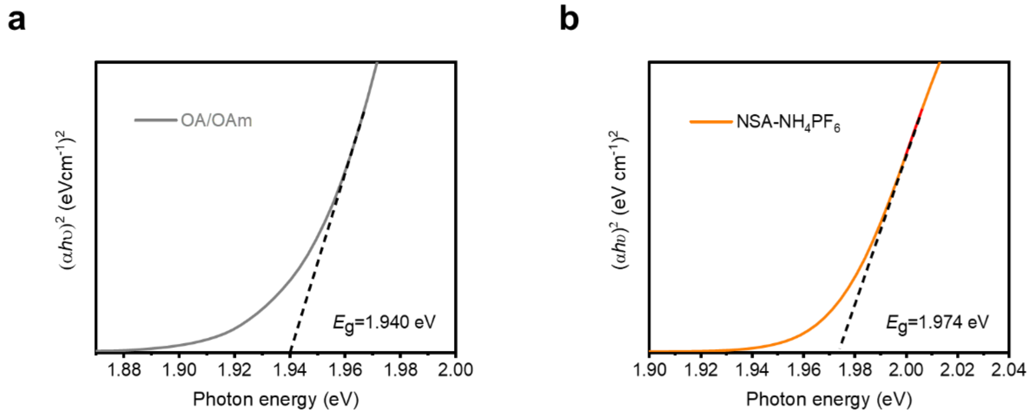

Supplementary Figure 15 Tauc plots of absorption spectra of different QD films. Tauc plots of $CsPbI_3$ QDs films treated with different ligands, (a) oleci acid/oleylamine (OA/OAm) QDs, (b) 2-naphthalenesulfonic acid hexafluorophosphate (NSA-NH_4PF_6) QDs.

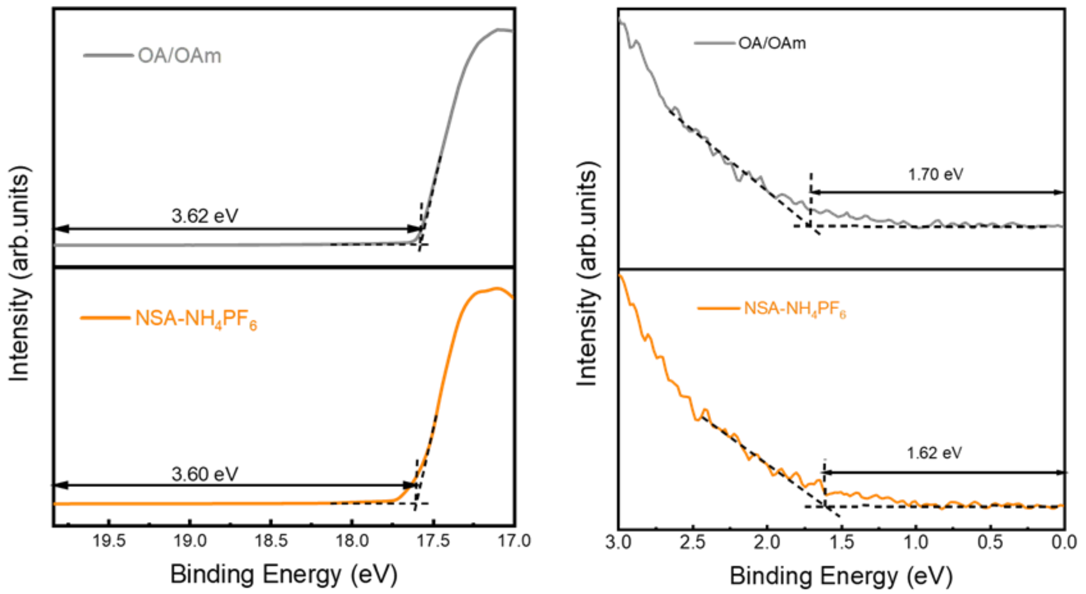

Supplementary Figure 16 Measured energy levels of different QD films. The

ultraviolet photoelectron spectroscopy (UPS) of oleic acid/oleylamine (OA/OAm) QD film and 2-naphthalenesulfonic acid hexafluorophosphate (NSA-NH_4PF_6) QD film.

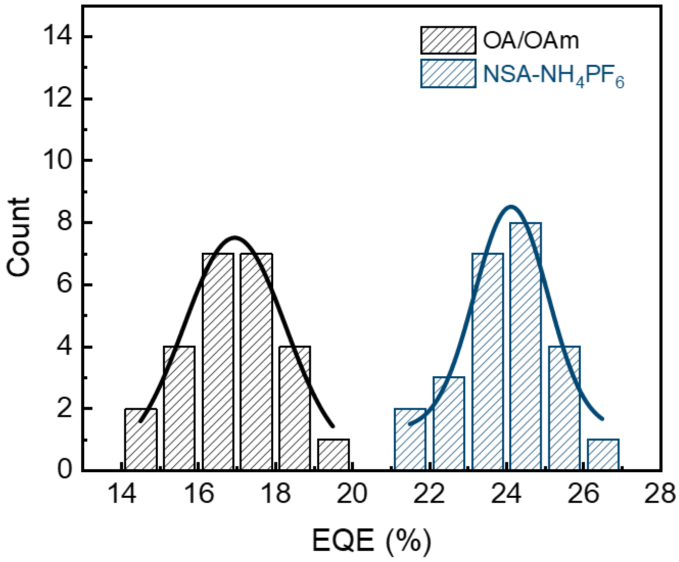

Supplementary Figure 17 Statistic of external quantum efficiency of different QD-based devices. Histograms of peak external quantum efficiency (EQE) values obtained from 25 light-emitting diodes (LEDs).

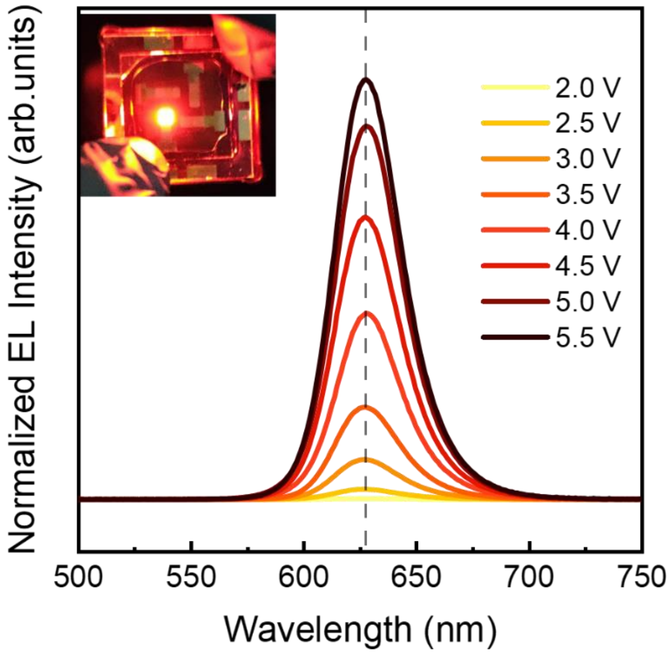

Supplementary Figure 18 Emission stability of devices driven at different voltages. Electroluminescence (EL) spectra of light-emitting diodes (LEDs) based on 2-naphthalenesulfonic acid hexafluorophosphate (NSA-NH_4PF_6) QDs at different operating voltages; the inset image shows the luminescence of the device driven at 5.5 V.

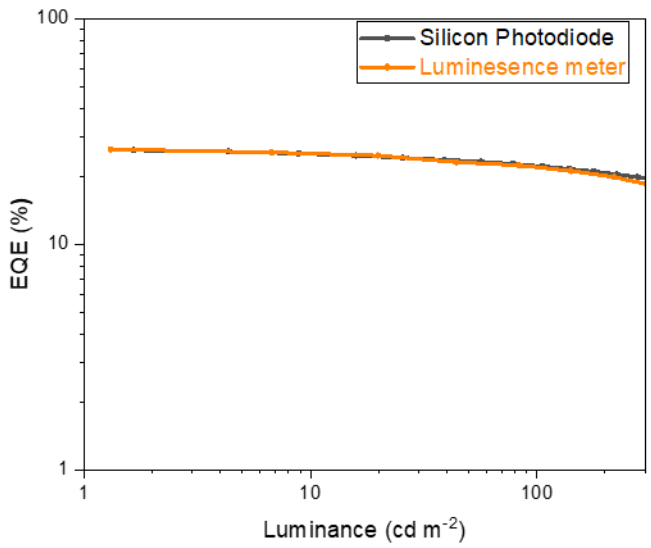

Supplementary Figure 19 calibration of device performance. Photodiode measured external quantum efficiency (EQE) calibration with a luminescence meter.

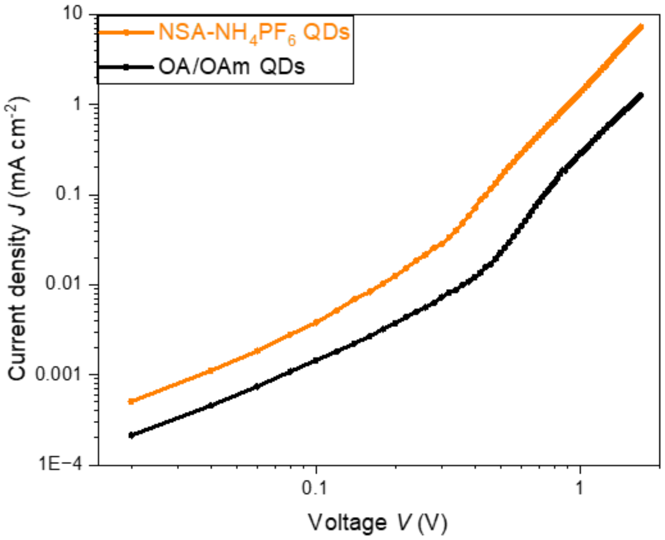

Supplementary Figure 20 ***J-V*** **curve of different QDs-based electron-only devices.**

J-V curve of the electron-only device for 2-naphthalenesulfonic acid hexafluorophosphate (NSA-NH_4PF_6) QDs and oleic acid/oleylamine (OA/OAm) QDs

Supplementary Table 1 Performance of reported PeLEDs. Performance summary of high-performance blue, green, and red LEDs based on perovskite colloidal QDs.

Color	EL (nm)	Emitting layer	EQE (%)	L_{max} (cd m^{-2})	T_{50} (min)	Ref.
Blue	483	(Cs/FA)$PbBr_3$ QDs	9.5	700	4.1 (100 cd m^{-2})	Nat. Photonics 13, 760-764 (2019)
Blue	479	$CsPbBr_3$ QDs	12.3	~450	20 (90 cd m^{-2})	Nat. Nanotechnol. 15, 668-674 (2020)
Blue	490	$CsPbBr_3$ QDs	14.6	403	12 (134 cd m^{-2})	Adv. Mater. 34, 2205092 (2022)
Green	516	$CsPbBr_3$ QDs	18.7	21000	30 (1000 cd m^{-2})	Nat. Commun. 11, 3902 (2020)
Green	511	$CsPbBr_3$ QDs	19.3	2030	25 (100 cd m^{-2})	Nat. Photonics 15, 379-385 (2021)
Green	~510	$CsPbBr_3$ QDs	22	~10000	60 (1200 cd m^{-2})	Nat. Nanotechnol. 15, 668-674 (2020)
Green	530	$FA_xGA_{1-x}PbBr_3$ QDs	23.1	~10000	64 (100 cd m^{-2})	Nat. Nanotechnol. 17, 590-597 (2022)
Green	531	$FA_xGA_{1-x}PbBr_3$ QDs	23.4	24000	132 (100 cd m^{-2})	Nat. Photonics 15, 148-155 (2021)
Red	645	CsPb(Br/I)$_3$ QDs	14.1	794	180 (100 cd m^{-2})	Nat. Photonics 12,681-687 (2018)
Red	620	MAPb(Br/I)$_3$ QDs	20.3	627	342 (100 cd m^{-2})	Nature 591, 72-77 (2021)
Red	653	CsPb(Br/I)$_3$ QDs	21.3	500	5 (100 cd m^{-2})	Nat. Photonics 12, 681-687 (2018)
Red	628	$CsPbI_3$	26.04	4203	729 (1000 cd m^{-2})	This work

Supplementary Table 2 Performance of reported red PeLEDs. Performance summary of high-performance red (EL < 650 nm) PeLEDs devices based on colloidal QDs, quasi-2D, and 3D films.

**Perovskite**	**EL (nm)**	**Max. EQE (%)**	**L_{max} (cd m^{-2})**	**T_{50} (min)**	**Reference**
$(PEA:NMA)_2Cs_{n-1}Pb_nI_{3n+1}$ Quasi-2D	635	12.4	1453	103 (100 cd m^{-2})	ACS Energy Lett. 6, 2386-2394 (2021)
$CsPb(Br/I)_3$ QDs	625	12.9	3382	-	Small 16, 2001062 (2020)
$CsPb(Br/I)_3$ QDs	645	14.1	794	180 (100 cd m^{-2})	Nat. Photonics 12, 681-687 (2018)
$CsPbI_{3-x}Brx$ film	638	17.8	9000	100 (100 cd m^{-2})	Sci. Adv. 8, eabq2321 (2022)
$MAPb(Br/I)_3$ QDs	620	20.3	627	342 (100 cd m^{-2})	Nature 591, 72-77 (2021)
$CsPbl_3$ QDs	636	20.8	3775	7.4 (110 cd m^{-2})	Nano Lett. 22, 8266-8273, (2022)
$CsPb(Br/I)_3$ QDs	637	21.8	6491	15 (120 cd m^{-2})	Adv. Funct. Mater. 2300116 (2023)
$CsPbl_3$ QDs	640	23.0	~1000	600 (200 cd m^{-2})	Angew. Chem. Int. Ed. 60, 16164-16170 (2021)
$CsPbI_3$ QDs	628	26.04	4203	729 (1000 cd m^{-2})	This work

Supplementary References

1. Kresse, G. & Furthmüller, J. Efficiency of ab-initio total energy calculations for metals and semiconductors using a plane-wave basis set. *Comput. Mater. Sci.* **6**, 15–50 (1996).
2. Kresse, G. & Hafner, J. Ab initio molecular dynamics for liquid metals. *Phys. Rev. B* **47**, 558–561 (1993).
3. Blöchl, P. E. Projector augmented-wave method. *Phys. Rev. B* **50**, 17953–17979 (1994).
4. Joubert, D. From ultrasoft pseudopotentials to the projector augmented-wave method. *Phys. Rev. B - Condens. Matter Mater. Phys.* **59**, 1758–1775 (1999).
5. Itaya, K., Uchida, I. & Neff, V. D. Electrochemistry of Polynuclear Transition Metal Cyanides: Prussian Blue and Its Analogues. *Acc. Chem. Res.* **19**, 162–168 (1986).
6. Pack, J. D. & Monkhorst, H. J. 'special points for Brillouin-zone integrations'-a reply. *Phys. Rev. B* **16**, 1748–1749 (1977).
